# Chemical Composition and Metabolomic Analysis of *Amaranthus cruentus* Grains Harvested at Different Stages

**DOI:** 10.3390/molecules27030623

**Published:** 2022-01-19

**Authors:** Tlou Grace Manyelo, Nthabiseng Amenda Sebola, Zahra Mohammed Hassan, Jones Wilfred Ng’ambi, William James Weeks, Monnye Mabelebele

**Affiliations:** 1 Department of Agriculture and Animal Health, College of Agriculture and Environmental Sciences, University of South Africa, Florida 1710, South Africa; manyelo.t.g@gmail.com (T.G.M.); sebolan@unisa.ac.za (N.A.S.); zahrabattal@gmail.com (Z.M.H.); 2 Department of Agricultural Economics and Animal Production, University of Limpopo, Sovenga 0727, South Africa; jones.ngambi@ul.ac.za; 3 Agricultural Research Services, Department of Agriculture and Rural Development, Potchefstroom 2520, South Africa; wjweeks123@gmail.com

**Keywords:** alternative grains, amaranth, chemical composition, harvest stage, nutrition

## Abstract

This study aimed at investigating the impact of early versus normal grain harvesting on the chemical composition and secondary metabolites of *Amaranthus cruentus* species grown in South Africa. Mature harvested grain had higher (*p* < 0.05) DM, CF, NDF and ADF content compared to prematurely harvested grain. There were no significant (*p* > 0.05) differences between CP, ADL and GE of premature and mature harvested grains. Mature harvesting resulted in higher grain Ca, P, Mg and K content. Essential amino acids spectrum and content remained similar regardless of maturity at harvest. The grains displayed an ample amount of unsaturated fatty acids; the highest percentage was linoleic acid: 38.75% and 39.74% in premature and mature grains, respectively. β-Tocotrienol was detected at 5.92 and 9.67 mg/kg in premature and mature grains, respectively. The lowest was δ-tocotrienol which was 0.01 and 0.54 mg/kg in premature and mature grains, respectively. Mature harvested grain had a higher secondary metabolite content compared to premature harvested grains. The results suggest that mature harvested *Amaranthus cruentus* grain contain more minerals and phytochemicals that have health benefits for human and livestock immunity and gut function, which ultimately improves performance. This study concludes that *A. cruentus* grown in South Africa is a potential alternative cereal to major conventional cereals.

## 1. Introduction

With the rising trend of the consumption of functional foods among the population, it is time to investigate alternative crops that can provide nutrients and health benefits at the same time. In addition, adverse climatic conditions have affected the yield of conventional crops, which warrants the search for alternative crops. Furthermore, the projected increase in the population to nine billion by the year 2050 [1] has put pressure on the agricultural sector to diversify the means of food production. Development and diversification of food and feed sources through investigation of currently unexploited crops capable of providing livestock and humans with alternative nutrients is essential. Amaranth is a highly nutritious pseudocereal crop with attractive nutraceutical properties [2,3]. It is a C4 plant that can tolerate harsh climatic conditions and plant diseases. The main cultivars used for grains include *Amaranthus hipochondriacus*, *Amaranthus cruentus* and *Amaranthus caudatus*. Amaranth grains contain a significant quantity of protein, which ranges between 14% and 17%, fat (5–9%) and starch (62%). However, Robertson and Clemants [4] reported the highest biological value of amaranth protein to be 75–79%. The essential amino acids in amaranth proteins are important for building new cells and tissues [5]. According to Januszewska-Jóźwiak and Synowiecki [6], Fidantsi and Doxastakis [7], and Paśko et al. [8], the protein of *A. cruentus* grains consists of albumins, globulins, prolamins and glutelins fractions that range from 48.9–65%, 13.7–18.1% and 1.0–3.2% to 22.4–42.3% of the total protein, respectively. In addition, it contains sulphur amino acids such as methionine and cysteine [9]. *Amaranthus* lipid content is also of interest because of its fatty acid profile that is characterised by three fatty acids, namely, palmitic, oleic and linoleic acids; the oil is highly unsaturated, containing more than 70% unsaturated fatty acid [10]. All these properties made the amaranth a promising alternative crop. *Amaranthus cruentus*, one of the three globally recognised grain amaranth types, has been identified as a prime candidate for pseudocereal developments.

The potential value of climate smart crops, such as amaranth, in terms of their inclusion in animal and human diets is becoming more apparent as global demand increases. Amaranth is considered a highly nutritious food crop with excellent scope in terms of the nutritional value derived from both grain and leaves [11]. Most often, amaranth is used as feed for animals; however, its endowment with abundant secondary metabolites, such as flavonoids, alkaloids, phenols, vitamins and macro- and microelements as well as polyunsaturated fatty acids, also makes it an excellent plant for human consumption. Metabolites are important for increasing the body’s resistance to infections, regulating blood pressure, blood biochemical parameters and improving the composition of gastrointestinal tract microflora populations that ensure gut health [12]. It should be stressed, however, that not all secondary metabolites have beneficial properties; phytochemicals, such as tannins, oxalates, phytates, trypsin inhibitors, saponins, and nitrates, are anti-nutritional factors found in amaranth. Anti-nutritional factors may have negative effects on the digestibility of amaranth-derived feed components that could indirectly impact animal productivity [13]. It is therefore imperative that the concentration of amaranth-derived feed components be carefully regulated and incorporated at reduced concentrations. Suitable processing methods should be employed in order to eliminate the anti-nutrients present in amaranth grain. Premature harvesting is a recognised practice for protecting grain yield and quality [14]. Mature amaranth inflorescences are prone to shattering by wind, and plants should not be left to dry in the field. The indeterminate flowering pattern resulting in an extended grain set period predisposes inflorescences to bird and insect predation with both yield and quality loss. Grain maturation may furthermore impact negatively on the nutritional profile, as the fibre-rich pericarp hardens around grains. Little empirical data are available on the impact that the harvesting period has on amaranth grain’s nutritional yield, despite the fact that extensive research on amaranth chemical and nutritional properties are available. The potential impact of the sap-sucking insect guild on grain yield and quality has been inadequately explored. Grains feeding *Heteroptera* and *Sternorrhyncha* (order *Hemiptera*), in particular, may occur in high abundance and diversity on pseudograin panicles including *A. cruentus* and *Cenopodium* quinoa in South Africa [15,16]. Although *A. cruentus* plant are leafy vegetables (*Morogo*) consumed in South Africa, the nutritional composition of the grains and the optimal time of harvesting has not been fully investigated. Determination of the optimal harvesting periods is imperative to the success of future industries. This current study aimed at being a first investigation as to the effect of harvesting stage on the nutritional and chemical composition of the grain amaranth, intended for human and animal nutrition.

## 2. Results

### 2.1. Proximate Analysis of Early and Normally Harvested Grains

#### Chemical Composition

Amaranth grain harvests are sensitive to both abiotic and biotic threats such as wind, bird and insect predation. Measures taken by producers should, however, not compromise the nutritive content of harvested grain. This is the most important reason for obtaining proximate analysis results for grain amaranth harvested at two developmental stages (Table 1). Mature harvested grain had higher (*p* < 0.05) DM, CF, NDF and ADF (90.60, 6.91, 11.08 and 7.24 g/100 g) contents than prematurely harvested grain (90.02, 5.77, 9.33 and 5.91 g/100 g). No similar results were reported on comparisons of DM, CF, NDF and ADF for prematurely and maturely harvested grain. No significant differences (*p* > 0.05) were observed between means for CP, ADL and GE calculated for premature and maturely harvested grains. During this study, crude protein was found to be within acceptable levels required for nutritional provision of poultry during both starter and grower diets. Significantly higher (*p* < 0.05) EE and ash contents (7.75 and 3.75 g/100 g, respectively) were found in mature grain compared to immature grain (6.33 and 3.13 g/100 g, respectively). Prematurely harvested grain had significantly higher (*p* < 0.05) starch content (37.96 g/100 g) compared to mature grain (29.11 g/100 g) (Table 2).

### 2.2. Mineral Composition

The mineral composition of premature and mature harvested amaranth grain is presented in Table 2. Mature harvested grain exhibited higher (*p* < 0.05) Ca, P, Mg and K contents (2771.07, 5024.56, 3501.36 and 5101.99 mg/kg, respectively) compared to premature harvesting (2125.41, 3966.24, 2805.53 and 4951.02 mg/kg, respectively). Prematurely harvested amaranth grain had a significantly higher (*p* < 0.05) Na content (46.99 mg/kg) when compared with mature grain (29.45 mg/kg). Significantly higher trace mineral content (*p* < 0.05) for Cu, Mn and Zn (6.95, 31.31 and 59.96 mg/kg, respectively) were present in premature harvested grain compared to mature grain (5.95, 23.71 and 49.97 mg/kg, respectively). Mature harvested grain contained significantly higher (*p* < 0.05) Fe (147.01 mg/kg) compared to prematurely harvested grain (104.97 mg/kg).

### 2.3. Amino Acid Profile

Amino acid composition of premature and mature harvested amaranth grain is shown in Table 3. The results of the present study showed that there were no significant differences (*p* > 0.05) between the essential amino acids and serine, glycine, glutamine, alanine, proline, isoleucine and phenylalanine content of premature and mature harvested grain. Premature harvested grain had a significantly higher (*p* < 0.05) aspartic acid content (0.71 g/100 CP) compared to mature grain (0.67 g/100 CP) (Table 3). Slight deviations in amino acid concentration were observed for a number of essential and non-essential amino acids (Table 3), but in none of the instances, except for Aspartic acid, were deviations significant.

### 2.4. Fatty Acids

The oil in the current study ranged between 3.9% and 6.4% in premature and mature *Amaranthus cruentus* grains, respectively. It contains mostly unsaturated fatty acids, which were seen to be almost similar in premature and mature grains. The dominant fatty acids in premature harvested grains were palmitic acid (15.15%), oleic acid (28.67%) and linoleic acid (38.75%). Whereas dominant fatty acids in mature harvested grains were palmitic acid (12.03%), oleic acid (30.65%) and linoleic acid (39.74%) (Table 4). The minority fatty acids reported in this study were myristic acid, linolenic acid, palmitoleic acid, arachidic acid and stearic acid. The current study showed a high percentage of unsaturated fatty acids (UFAs): 68.29% and 70.97% in premature and mature grain, respectively. Saturated fatty acid (SFA) percentages were 13.76% and 18.39% for mature and premature grain, respectively. The ratios of saturated to unsaturated fatty acids were 0.22 and 0.27 for mature and premature grain, respectively.

### 2.5. Tocopherols and Tocotrienols

**Figure 1:**
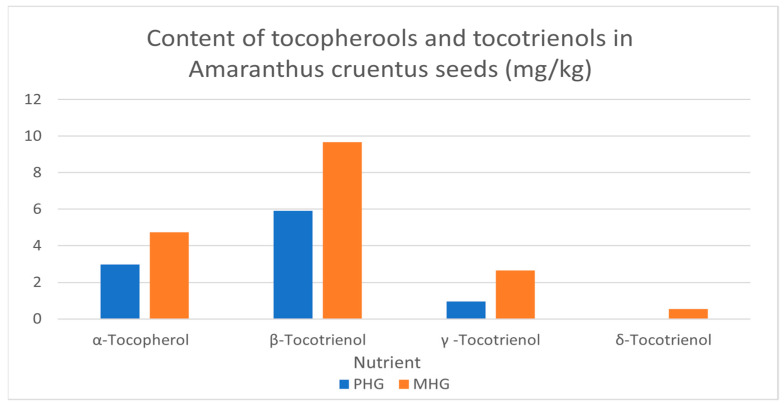
The content of tocopherols and tocotrienols in *Amaranthus cruentus* grains, mg/kg. PHG: premature harvested grain; MHG: mature harvested grain.

Tocopherols and tocotrienols are lipid soluble compounds. In this study, few tocols were detected in the *Amaranthus cruentus* grains, and the most dominant was β-tocotrienol (5.92 and 9.67 mg/100 g) in premature and mature grain, respectively. The lowest was δ-tocotrienol which was (0.01 and 0.54 mg/100 g) in premature and mature grain, respectively (Figure 1).

### 2.6. Secondary Metabolites

**Figure 2:**
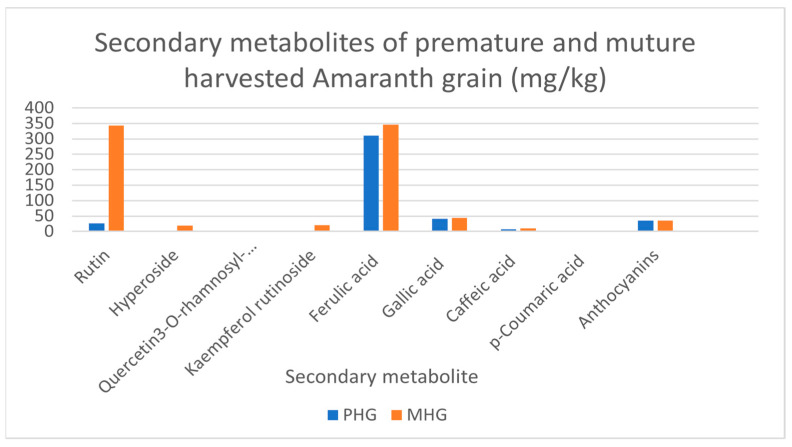
Secondary metabolites in premature and mature *Amaranthus cruentus.* PHG: premature harvested grain; MHG: mature harvested grain.

Secondary metabolites present in premature and mature harvested amaranth grain are shown in Figure 2. Mature grain contained significantly higher (*p* < 0.05) concentrations of rutin, hyperoside, tryptophan, quercetin 3-*O*-rhamnosyl-rhamnosyl-glucoside and kaempferol rutinoside (342.20, 18.49, 39.90, 0.96 and 19.99 mg/kg, respectively) compared to premature harvested grain (26.30, 0.00, 27.34, 0.00 and 1.82 mg/kg, respectively). On the other hand, the content of phenolic acids, such as ferulic acid, gallic acid, caffeic acid, *p*-coumaric acid and anthocyanins, was 310, 41.0, 6.5, 1.2 and 35.2 mg/kg, respectively, for premature grains and 345.20, 43.48, 9.93, 2.94 and 35.92 mg/kg, respectively, for mature grains. Ferulic acid content was significantly higher (*p* < 0.05) in the mature grain. Figure 2 illustrates the contents of secondary metabolites detected in early and normally harvested amaranth grain. Rutin was determined in premature harvested grain, whereas hyperoside, quercetin 3-*O*-rhamnosyl-glucoside and kaempferol rutinoside were not determined. However, these metabolites were detected but not quantified in the analysis. Normally harvested grain showed all secondary metabolites with a clear indication of the abundance of ferulic acid followed by rutin. Small contents of hyperoside and kaempferol rutinoside were observed.

## 3. Discussion

In this study, the protein content of *Amaranthus cruentus* was more than 12%. Both prematurely and maturely harvested grain can confidently be used as a good source of protein. CF, NDF and ADF were significantly higher in mature harvested grain than prematurely harvested grain. To the best of our knowledge, no similar results have been reported on comparisons of CF, NDF and ADF for prematurely and maturely harvested grains. However, high fibre content was expected in mature grain due to the increased lignin, hemicellulose and cellulose, which are constituents of fibrous tissue essential for grain coat structure [17]. Dietary fibre may positively impact on animal gut health by increasing satiety, improving behaviour and overall well-being. Fibre is an essential dietary component for maintaining normal physiological functionality in the digestive tract [18]. Plant-based materials providing more than 12% of their calorific value from protein are categorised as good sources [19].

With regards to the lipid content, Soriano-García et al. [20] reported lower lipids and ash content values for mature and prematurely harvested amaranth grain compared to those reported in this study. Differences may be ascribed to environmental conditions during production and amaranth type, which may account for differences in nutritional yield. Prematurely harvested grains had higher starch content (37.96 g/100 g) compared to mature grain (29.11 g/100 g). Starch content reported for amaranth grain in the literature ranges between 48 and 69 g/100 g [21], which is higher than that of the current study in both the premature and mature harvested grains. Moreover, De Bock et al. [22] and Li et al. [23] reported that quinoa had great variation in starch content (58.2–67.6 g/100 g) compared to the current study. Environmental conditions leading to plant stress may have been a dominant factor resulting in the lower-than-expected starch content. Results indicate the potentially important nutritional yield impact that grain maturity has at the harvesting stage. Mature harvested grain exhibited higher Ca, P, Mg and K contents (2771.07, 5024.56, 3501.36 and 5101.99 mg/kg, respectively) compared to premature harvesting (2125.41, 3966.24, 2805.53 and 4951.02, mg/kg respectively). The findings of the present study showed that they were in close agreement with the results reported by De Bock et al. [22]; however, there were lower amounts of minerals compared to the values of the present study and significantly higher values of minerals in a study by Li et al. [23]. The minerals reported in this study are essential to the formation and maintenance of healthy skeletal structures [24]. According to Firman [25], the recommended daily intake of Ca, P, Mg and K in poultry is 3.750, 350, 55 and 165 mg per day, respectively, as influenced by rearing phase, sex and physiology [26]. Both premature and mature harvested grains can be a good source of P, Mg and K.

The results of this study indicate that both premature and mature harvested grains can supply adequate Fe to address deficiencies encountered in human and livestock. The findings also confirm the fact that amaranth grain can confidently be recommended to address the national and global issue pertaining to high animal feed cost. In addition, sodium (Na) is known to be a constituent of common salt, and it serves as an important osmotic regulator in body fluids [27]. The recommended daily allowance (RDA) for chicken Na would be 165 mg [25]. Premature and mature harvested grains in the present study may be inadequate in providing the recommended dietary intake of Na. The amino acids contents were more or less similar in both premature and mature *Amaranthus* grains. The probable reason for the observed similarity in the content of amino acids may be that amino acids as building blocks for proteins are central to and present during all developmental growth stages. The values reported in the present study agree with the results reported by Soriano-García et al. [20]. Aspartic acid is a non-essential amino acid central to hormone production and release as well as nervous system functionality in both human and animals [28]. The spectrum of amino acids contained in amaranth grain was unaffected by maturity at harvest. The oil of *Amaranthus* is mostly extracted from the grain of *A. cruentus* and *A. hipochondriacus*, and the oil content ranges between 4.8% and 8.1% [29,30]. The results of the current study are in agreement with the results obtained by He and Corke [30] and Hlinková et al. [31]. The linoleic acid content reported in this study was within the range of the results obtained by Hlinková et al. [31], who reported a range of 33.3–38.7% for *Amaranthus cruentus*. The consumption of unsaturated fatty acids reduces the low-density lipoprotein levels in blood and reduces the probability of cardiovascular diseases in both human and animals [32]. The high percentage of unsaturated fatty acids in premature and mature grains is in agreement with the results reported by Nasirpour-Tabrizi et al. [10]. The ratios of saturated to unsaturated fatty acids for mature and premature grains were within the range reported by El Gendy et al. [33] and He et al. [34]; however, the ratios were lower than the 0.61% reported by Gresta et al. [35] in *A. cruentus* grown in a Mediterranean environment. *A. cruentus* can be recommended for human and animal health due to the fact of its high content of balanced and favourable fatty acids.

The tocopherols were within the range reported by Lehmann et al. [36] on a study that investigated different amaranth species, including *Amaranthus cruentus* [37], and also reported on the endowment of *A. cruentus* grains with tocopherols. β-Tocotrienol and δ-tocotrienol were reported to have the ability to inhibit the activity of HMG-CoA reductase, while α-tocopherol is known for its ability to increase reductase activity in both humans and animals [38].

The results of the present study agree with findings reported by Karamać et al. [39], who reported high flavonoid concentrations in mature grain with rutin being the highest. The presence of phenolic compounds could primarily be responsible for the antioxidant activity of amaranth in early flowering and grain fill stages [39]. According to Kaur and Muthuraman [40], rutin attracted attention in animal nutrition circles due to the fact of its anti-inflammatory qualities. Growing evidence supports claims of the benefits of plant-based flavonoids, such hyperoside, to animal health mediated through immuno-stimulatory, antioxidant, anti-inflammatory, and antimicrobial properties [12]. According to Kamboh et al. [12], flavonoids improve immunity and haematological indices in poultry and shows potential as an alternative for synthetic antibiotics. Tryptophan is an essential dietary component central to protein synthesis as a precursor to biologically active compounds such as serotonin, melatonin and quinolinic acid. The presence of ferulic acid in the diet is believed to be beneficial for health, because it aids in lowering the cholesterol, anti-thrombosis, anti-inflammatory and anti-cancer in human and animals [41]. The mature grain of *Amaranthus cruentus* proved to have ample ferulic acid content, which makes it a preferred candidate when selecting it for human and animal health purposes. Several studies indicated that daily intake of tryptophan in a supplemented diet maintains physiological processes such as tissue synthesis, feed uptake, growth, feed conversion ratio (FCR) and immunity enhancement in broiler chickens [12,42]. According to de [43], a daily tryptophan intake of 37 mg/kg is recommended in broiler chickens. This requirement can only be met through inclusion of mature harvested amaranth grain. Quercetin and kaempferol rutinoside promote growth performance, oxidation stability, egg and meat quality and immune and anti-inflammatory responses (reduces arthritic inflammation) [44]. Quercetin is also believed to play a critical role in the inhibition of hydrogen peroxide-induced cataracts [45]. The average daily quercetin requirement was estimated at 15 mg/kg and approximated to 60 mg/kg per day for high concentration chicken production [46]. Both premature and mature harvested grain in the current study had quercetin concentrations below recommended daily intake for broiler chickens. Mature plant tissue had high flavonoid concentrations that positively altered the fatty acid profiles of meat and eggs by reducing cholesterol and triglyceride contents [12]. According to Lin et al. [47], the content of flavonoids (mainly rutin) increases during the growth cycle, and these compounds could be primarily responsible for the antioxidant activity of matured grains.

## 4. Materials and Methods

### 4.1. Harvesting of Amaranth Grains

*A. cruentus* grain was sampled from strip plantings at the Taung Experimental Farm (25°62′00′′ S, 27°98′00′′ E) in the Ruth Segomotsi Mompati District of the North West Province, South Africa. Planting was conducted through direct sowing in a sandy soil. Average temperatures at the sampling site were above 22 °C during summer and below 20 °C in winter. Amaranth was sown on 6 November 2019 (Austral spring) under dry land conditions (germinated with back-up irrigation) that received an average annual rainfall of less than 250 mm. Dual-stage grain sampling was performed from main inflorescences and related to the two-digit phenological code of the Biologische Bundesanstalt, Bundesortenamt and Chemical Industry (BBCH) scale [10,40]. Early sampling was conducted on 23 January 2020 when grains became available at the mid-milky stage (BBCH code: 75) (Table 1), and sampling of mature grain (BBCH code: 89) (Table 1) was conducted on 6 March 2020.

Grains samples were dried separately at room temperature in a well-ventilated laboratory and hammer milled before sifting through a 1 mm sieve into flour in preparation for chemical analyses as described.

### 4.2. Chemical Analysis

Proximate analysis for moisture, ash, crude protein (N × 6.25), fat and starch were carried out according to the standardised methods of the AOAC [48]. Grain flour samples were oven-dried and weighed prior to being ashed in a muffle furnace at 550 °C for 6 h. The ash was acid digested by adding 1 mL 55% (*v/v*) HNO_3_. Calcium, magnesium, manganese, zinc, iron, sodium, potassium, copper, sulphur and phosphorus concentrations were determined via the AOAC method (i.e., 6.1.2 AOAC) [48] after a cooling period. This process involved inductively coupled plasma spectroscopy. Neutral detergent fibre (NDF) and acid detergent fibre (ADF) were determined. The gross energy content of the milled samples was determined with adiabatic bomb calorimetry (Gallenkamp, Autobomb, London, UK). Fat- and ether-extracted lipid contents were estimated using TecatorSoxtec.

### 4.3. Amino Acid Profile

Amino acid separation and detection was performed via a Waters Acquity Ultra Performance Liquid Chromatograph (UPLC), fitted with a photodiode array (PDA) detector. Derivatising agent (AQC) was prepared by addition of 1 mL of dry acetonitrile to the reagent—a vial containing 3 mg of AQC—the vial was then heated, vortexed and sonicated to ensure the reagent dissolved completely.

This required 1 µL of sample/standard solution to be injected into the mobile phase, which conveyed derivatised amino acids onto a Waters UltraTax C 18 column (2.1 × 50 mm × 1.7 µm) maintained at 60 °C. Elution of analytes off the column was performed by running a gradient. Analytes eluting off the column were detected by the PDA detector with individual amino acids coming off the column at unique retention times.

### 4.4. Oil extraction and Fatty Acids Determination

The oil content of the *Amaranthus cruentus* grain was extracted using the method of STN 461011-28 (1988). Triplicate of the grain samples were extracted using a 100 mL of n-hexane in a Soxhlet. The first extraction lasted 5 h at 50 °C temperature, and then the grain was dried and extracted for the second time for 4 h. The formula used was ((m2 − m1/m) × 100) × (100/W), where m2 is the mass of the flask with isolated oil, m1 is the mass of the empty flask; m is the mass of the weighed sample (g), and W is the dry weight (%). Methyl esters were prepared by mixing 10 mg of oil with 1 mL of n-hexane and 0.1 trans-esterificating agents (sodium methanolate in cyclohexane). In 20 min, 13% methanolic HCl (0.5 mL) was added. The solution was centrifuged at 850× *g* for 5 min at ambient temperature (20–25 °C). Analysis of fatty acids was performed using gas liquid chromatography (GLC) to analyse the fatty acids, fitted with a photodiode array (PDA) detector. The quantification was performed according to similar individual fatty acids in the chromatograms.

### 4.5. Determination of Tocopherols and Tocotrienols

A Waters Acquity UPLC was fitted with a PDA detector and was used to determine the composition of tocols in the *Amaranthus cruentus* grains.

### 4.6. Determination of Secondary Metabolites

Extracts were prepared with 2 g of dry grain material +15 mL of 50% methanol and 1% formic acid dissolved in water with ultrasonication for 1 h and left standing overnight. This was followed by centrifugation and transferral of the supernatant to a glass vial readied for LC-MS analysis. Samples were then analysed via the LC-MS method using a Waters SYNAPT G2 Quadrupole time-of-Flight (QTOF) mass spectrometer (MS) connected to a Waters Acquity UPLC (Waters, Milford, MA, USA) for high-resolution UPLC-MS analysis. Electrospray ionisation was used in the negative mode with a cone voltage of 15 V, desolvation temperature of 275 °C and desolvation gas at 650 L/h; the rest of the MS settings were optimised for the best resolution and sensitivity. Data were acquired by scanning from 150 to 1500 *m/z* in the resolution mode as well as in the MSE mode.

### 4.7. Statistical Analysis

Data were subjected to one-way ANOVA performed with SAS [49] software. The general linear model employed was:

Y_ijk_ = µ + SM_i_ + E_ij_

where Y_ijk_ is the observation of the dependent variable ijk (i.e., chemical components of grain), µ is the fixed effect of the population mean for the variable, SM_i_ is the phenological stage of harvested grain (i = 2; premature and mature), and E_ij_ is the random error associated with the observation of ij, assumed to be normally and independently distributed. Where significant differences were observed, means separation was conducted by LSD test at a 5% significance level. Pearson’s correlation coefficient was calculated to determine the relationship between phenolic compounds with chemical and mineral compositions.

## 5. Conclusions

Amaranth is not only a climate smart crop, but it also has favourable physical characteristics and chemical composition regardless of the stage of cultivation. Amaranth grain is underutilised in spite of its unique properties, high nutritional yield and high competitiveness comparative to mainstream grains. The present study confirms the invaluable contribution of amaranth grain, as a source of energy, starch, protein, fibre, vitamins E isomers, unsaturated fatty acids, and essential secondary metabolites, to the nutrition and health of humans and animals alike. The harvesting stage of amaranth grain will have an effect on some of its nutritive value and quality. The results of this study indicated that mature harvested grain had, overall, outstanding nutritional quality, even though amino acid composition was not affected by harvesting maturity. Secondary metabolites, specifically flavonoids, were shown to be abundant in mature harvested grains, which highlights its potential for enhancing immunity and gut functions in human and animals. *Amaranthus cruentus* should be accorded significant attention as a prime candidate for the replacement of conventional grains in human and animal diets.

## Figures and Tables

**Table 1:** Proximate analysis composition of premature and mature harvested grain amaranth (g/100 g).

Nutrient	Grain Maturity		SEM	Probability
	PHG	MHG		
DM	90.02	90.60	0.050	0.015
CP	18.16	18.26	0.050	0.293
CF	5.77 ^b^	6.91 ^a^	0.050	0.004
NDF	9.33 ^b^	11.08 ^a^	0.050	0.002
ADF	5.91 ^b^	7.24 ^a^	0.050	0.003
ADL	2.07	1.99	0.050	0.375
GE	17.47	17.55	0.050	0.375
EE	6.33 ^b^	7.75 ^a^	0.050	0.003
Starch	37.96 ^a^	29.11 ^b^	0.050	0.000
Ash	3.13	3.75	0.050	0.013

Values are the means of duplicate analysed grain amaranth samples. ^a,b^ Means followed by the same superscript in a row were not significantly different (*p* > 0.05). PHG: premature harvested grains MHG: mature harvested grain; SEM: standard error of the mean.

**Table 2:** Mineral composition of premature and mature harvested amaranth grain (mg/kg).

Nutrient	Grain Maturity		SEM	Probability
	PHG	MHG		
Macro-Minerals				
Calcium	2125.41 ^b^	2771.07 ^a^	0.05000	0.0001
Phosphorus	3966.24 ^b^	5024.56 ^a^	0.05000	0.0001
Magnesium	2805.53 ^b^	3501.36 ^a^	0.05000	0.0001
Potassium	4951.02 ^b^	5101.99 ^a^	0.03536	0.0001
Sodium	46.99 ^a^	29.45 ^b^	0.05000	0.0001
Trace Minerals				
Copper	6.95 ^a^	5.95 ^b^	0.05000	0.0050
Manganese	31.31 ^a^	23.71 ^b^	0.05000	0.0001
Iron	104.97 ^b^	147.01 ^a^	0.05000	0.0001
Zinc	59.96 ^a^	49.97 ^b^	0.05000	0.0001

Values are the means of duplicate analysed amaranth grain samples. ^a,b^ Means followed by the same superscript in a row were not significantly different (*p* > 0.05). PHG: premature harvested grain; MHG: mature harvested grain; SEM: standard error of the mean.

**Table 3:** Amino acid composition of premature and mature harvested grain (g/100 g CP).

	Grains Maturity			
	PHG	MHG	SEM	Probability
Essential Amino Acids				
Histidine	2.41	3.21	0.050	0.049
Arginine	5.60	5.66	0.050	0.001
Threonine	3.71	3.67	0.050	0.058
Lysine	3.22	4.18	0.050	0.058
Tyrosine	0.56	0.46	0.050	0.036
Methionine	2.21	2.26	0.050	0.167
Valine	2.18	2.28	0.050	0.003
Leucine	3.10	3.99	0.050	0.017
Non-Essential Amino Acids				
Serine	4.41	4.48	0.050	0.049
Glycine	3.60	1.60	0.050	0.009
Aspartic acid	4.71	4.87	0.050	0.058
Glutamine	11.22	11.18	0.050	0.058
Alanine	3.56	3.46	0.050	0.036
Proline	2.21	2.26	0.050	0.167
Isoleucine	2.18	2.28	0.050	0.0003
Phenylalanine	1.00	0.99	0.050	0.017

Values are the means of duplicate analysed amaranth grain samples. Means followed by the same superscript in a row were not significantly different (*p* > 0.05). PHG: premature harvested grain; MHG: mature harvested grain; SEM: standard error of the mean.

**Table 4:** Fatty acid composition of premature and mature harvested amaranth grain (%).

	Grain Maturity			
Fatty Acid Composition	PHG	MHG	SEM	Probability
Saturated Fatty Acids				
Myristic acid (C14:0)	0.16	0.16	0.000	0.0001
Palmitic acid (C16:0)	15.15	12.03	0.000	0.0001
Stearic acid (C18:0)	2.52	2.02	0.007	0.0001
Arachidic acid (C20:0)	0.56	1.55	0.007	0.0001
Unsaturated Fatty Acids				
Linoleic acid (C18:2)	38.75	39.74	0.000	0.0001
Linolenic acid (C18:3)	0.68	0.55	0.010	0.0001
Palmitoleic acid (C16:1 cis)	0.18	0.03	0.000	0.0001
Oleic acid (C18:1)	28.67	30.65	0.010	0.0001
Saturated	18.39	15.76	0.010	0.0001
Unsaturated	68.29	70.97	0.000	0.0001
Saturated/unsaturated	0.27	0.22	0.007	0.0001

Values are the means of duplicate analysed amaranth grain samples. PHG: premature harvested grain; MHG: mature harvested grain; SEM: standard error of the mean.

## Data Availability

Data available upon request to corresponding author: mabelm@unisa.ac.za.

## Sample Availability

Samples of the compounds of *Amaranthus cruentus* are available from the authors.
